# Use and quality of point-of-care microscopy, urine culture and susceptibility testing for urinalysis in general practice

**DOI:** 10.1080/02813432.2021.2022349

**Published:** 2022-01-13

**Authors:** Ida Kollerup, Anne Kathrine Aagaard Thomsen, Jette Brommann Kornum, Kirsten Inger Paulsen, Lars Bjerrum, Malene Plejdrup Hansen

**Affiliations:** a Center for General Practice, Aalborg University, Aalborg, Denmark; b Department of Clinical Microbiology, Aalborg University Hospital, Aalborg, Denmark; c Section of General Practice and Research Unit for General Practice, Department of Public Health, University of Copenhagen, Copenhagen, Denmark

**Keywords:** Urinary tract infections; microscopy; culture media; point-of-care testing; general practice; microbiological diagnosis

## Abstract

**Objective:** To describe the use and quality of point-of-care (POC) microscopy, urine culture and susceptibility testing performed in general practice in Northern Denmark from 2013 to 2018.

**Design:** Descriptive study

**Setting:** General practices receiving a fee for examining urine samples.

**Subjects:** Simulated urine samples containing uropathogenic bacteria distributed by the organisation for improvement of microbiological quality (MIKAP).

**Main outcome measures:** Percentage of use and correct answers for microscopy, culture and susceptibility testing.

**Results:** A total of 5361 samples were analysed by the use of microscopy (39.7%), culture (66.0%) and/or susceptibility testing (76.5%). For culture, Flexicult SSI urinary kit^tm^ (87.6%) demonstrated the highest percentage of correct answers followed by chromogenic agar (85.1%) and 2-plate dipslide (85.2%). Mueller Hinton agar with tablets had the highest percentage of correct answers for susceptibility testing of most bacterial strains (84.6%), followed by Flexicult (77.2%). Furthermore, susceptibility testing with tablets (range: 76.1–84.6%) was found to be more accurate than discs (range: 72.9–75.5%). Overall, the highest percentage of correct answers was obtained when examining urine samples containing *Escherichia coli*: Microscopy (78.3%), culture (87.0%) and susceptibility testing (range: 84.3–90.7%).

**Conclusion:** The quality of POC testing in general practice was high when examining urine samples containing the most common uropathogen *E. coli.* Surprisingly, susceptibility testing was more frequently used than culture. This approach may compromise the treatment decision as only cultures contribute with information about the flora composition and bacterial quantification. Interestingly, microscopy was the least used method even though the result may be reached within a few minutes.

**Key points**
- The quality of POC tests (microscopy, urine culture, susceptibility testing) performed in general practice was high when examining urine containing *E. coli*, whereas difficulties were observed for samples including *S. saprophyticus* or *K. pneumoniae.*
- Susceptibility testing was more often performed than urine culture, which indicates a problem as only urine cultures contribute with information about the flora composition and bacterial quantification.

## Introduction

General practice is responsible for about 75% of all antibiotics prescribed in Denmark, of which a urinary tract infection (UTI) is one of the most frequently stated indications [1,2]. The most common uropathogen identified in patients with UTI is *Escherichia coli,* which account for approximately 75–95% of cases [3].

Unfortunately, an increase in the use of antibiotics worldwide has resulted in emerging bacterial resistance [4]. Despite the lower use of antibiotics in Denmark, compared to other European countries, resistance of especially *E. coli* is observed [5]. A Danish study from 2017, investigating isolates from patients with suspected UTI in general practice, found that 45% of *E. coli* isolates to be resistant to at least one type of antibiotics commonly used in general practice [6]. Furthermore, a direct link between cumulative exposure to any antibiotic and the risk of developing a community-acquired infection due to third generation cephalosporin resistant *E. coli* was found in a recently published study [7]. The risk of developing an infection caused by resistant bacteria was highest during the first 12 months after exposure, with a decreasing trend over time, emphasising the possibility of resistant strains becoming susceptible again [7]. In order to reduce the unnecessary use of antibiotics the World Health Organisation (WHO) has advised enhanced use of diagnostic tests [8].

Only about 60% of patients presenting with typical symptoms of UTI, such as pollakisuria and dysuria, actually have a UTI [9]. Thus, treatment based on symptoms alone might lead to overuse of antibiotics. In Denmark, guidelines recommend that when patients are presenting with typical symptoms of UTI, a urine examination is indicated before prescribing antibiotics [10].

Point-of-care (POC) tests such as dipstick, phase-contrast microscopy (in the following called microscopy), urine culture and susceptibility testing are widely performed in-house and results are available, while the patient is in the consultation (dipstick, microscopy) or within 24 h (urine culture, susceptibility testing) [11,12]. When urine samples are sent for urinalysis at the microbiological laboratory, it may take up to three days to receive the results. Delay of microbiological testing increases the risk of initiating inappropriate antibiotic treatment, as the general practitioner (GP) prescribes antibiotics to patients presenting with typical symptoms of UTI before the test result is obtained [13]. Thus, rapid tests such as POC urine culture can be beneficial for reducing inappropriate prescribing of antibiotics.

Although POC tests have been used in Danish general practice for a number of years, only a few studies have investigated the quality of microscopy, urine culture and susceptibility testing when performed in general practice [14–18]. Generally, a high accuracy of POC tests has been found, but the overall quality of urine analysis performed in general practice by means of POC tests has not yet been evaluated and compared [16].

The aim of this study was to describe the use and quality of urinalysis performed in general practices in the North Denmark Region during 2013–2018 by use of POC microscopy, urine culture and susceptibility testing.

## Materials and methods

### Setting and recruitment

In 2002, the organisation for improvement of microbiological quality (MIKAP) was established to conduct quality control of microbiological analyses performed in Danish general practices. All practices receiving fees for examining urine samples are obliged to participate in the MIKAP quality control programme. This study was exclusively based on data collected from general practices located in the North Denmark Region, and data from a six-year period was analysed (2013–2018).

### Test material

Every calendar year, six urine samples, with standardised uropathogenic bacteria, were distributed to general practice for examination. The standardised strains were identical for all the participating practices. The strains differed each year but included common uropathogenic bacteria; *E. coli, Staphylococcus saprophyticus, Proteus mirabilis, Klebsiella pneumoniae, Enterococcus faecalis* and *Enterobacter cloacae*. The simulated urine samples included ≥10^5^ bacteria per mL to ensure confluent growth and were delivered in boric acid transport media. Furthermore, the general practice staff was instructed to either refrigerate (maximum 48 h) or examine the samples immediately after arrival.

Simulated urine samples were also sent for analysis at the Department of Clinical Microbiology (KMA) at the Aalborg University Hospital and to three other reference laboratories (control tests). For urine culture, the reference laboratories used either a biplate (consisting of a non-selective chromogenic agar and a medium selective for gram-positive bacteria) or a non-selective chromogenic agar and a non-selective blood agar. For susceptibility testing, they used Mueller Hinton agar with discs or tablets.

### Point-of-care tests

All participating general practices were asked to use their routine test for urinalysis. For urine culture either chromogenic agar, dipslide or Flexicult SSI urinary kit™ (mentioned as Flexicult onwards) were used. For susceptibility testing, either Flexicult, Mueller Hinton agar with tablets/discs or Iso-Sensitest with tablets/discs were used. The POC tests were performed in accordance with existing recommendations [11,15,19,20]. At least four types of the following antibiotics were included in the susceptibility testing: trimethoprim, sulfamethizole, ampicillin, nitrofurantoin, mecillinam or ciprofloxacin. Urinalysis was conducted by the general practice staff that normally perform laboratory work in the practice (GP or practice staff). After each sample was examined a short questionnaire was filled including:

- Microscopy: Number of bacteria per visual field at 400 times magnification and bacterial morphology (cocci arranged in clusters or chains, rods or a mixed flora)
- Urine culture: Method used, quantification of bacteria and mixed or single-strain culture
- Susceptibility testing: Method used and antibiotic resistance of the bacterial strain.

### Data analysis

Data was entered into Excel worksheets, and samples with any missing data were excluded. In order to describe the quality of urinalysis performed in general practice by use of POC tests, the results were calculated in percentage of correct answers, and 95% confidence intervals were noted.

In order to evaluate the quality when using various methods, the following assessment was used:

- Microscopy: Correct finding of ≥1 bacterium per visual field and correct bacterial morphology
- Urine culture: Correct finding of ≥10^5^ bacteria per mL and single-strain culture
- Susceptibility testing: Correct analysis of susceptibility testing of at least four types of antibiotics.

Following exceptions were agreed on due to an observed lower amount of bacteria per mL in the control tests performed by reference laboratories. This could be due to either a mistake at their preparation or if the concentration of boric acid had been too high.

- Microscopy: 0–1 bacterium was accepted for *P. mirabilis* distributed in 2017
- Urine culture: 10^4^ bacteria per mL was accepted for *E. coli* resistant to trimethoprim, sulfamethizole and ampicillin distributed in 2016.

Furthermore, a subgroup analysis focusing on the urinalysis quality when using various methods used for urine culture (Flexicult SSI urinary kit^TM^, chromogenic agar, 2-plate dipslide and 3-plate dipslide) and susceptibility testing (Flexicult SSI urinary kit^TM^, Mueller Hinton agar and Iso-Sensitest) was performed and results noted as percentage of correct answers.

### Ethics and data protection

The project was registered at the Center for General Practice at Aalborg University (ID 96-5). According to Danish law, ethical approval is not necessary for this type of study. Data was handled confidentially and anonymised in accordance with the Danish Data Protection Act.

## Results

**Figure 1.**
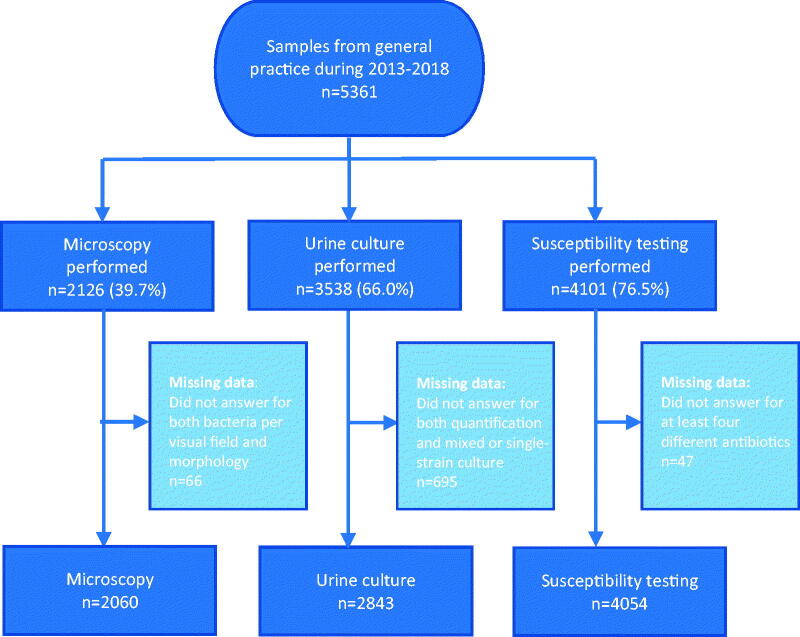
Attrition flow chart of urinalysis performed by use of microcopy, urine culture and susceptibility testing in general practice during 2013–2018.

On average, 149 (range: 132–168) general practices in the North Denmark Region attended the quality control programme each year from 2013 to 2018. A total of 5361 samples were collected during the study period and analysed by use of microscopy (39.7%), urine culture (66.0%) and/or susceptibility testing (76.5%). Figure 1 provides an overview of the dataset.

Tables 1 and 2 show the percentages of correct answers for microscopy and urine culture, respectively*. E. coli* (all strains) was answered correctly by microscopy for 78.3% of the samples. The lowest percentages of correct answers were found for samples containing *K. pneumoniae* (57.0%) and *S. saprophyticus* (30.7%). With regard to urine culture, the highest percentages of correct answers were found for *P. mirabilis* (88.2%), *E. faecalis* (87.7%) and *E. coli* (all strains) (87.0%). The lowest percentage of correct answers was observed for *K. pneumoniae* (72.9%). Concerning susceptibility testing, the highest percentage of correct answers was seen for *E. coli* (res: ampi + meci) (90.7%), and the lowest percentages were found for *E. cloacae* (67.1%) and *K. pneumoniae* (69.2%) (Table 3).

**Table 1.** Percentages of correct answers for microscopy.

Microscopy (*n*)	Correct answers (%) (95% CI)
*P. mirabilis* (229)	66.8 (60.3–72.9)
*E. faecalis* (340)	64.1 (58.8–69.2)
*E. cloacae* (112)	72.3 (63.1–80.4)
*K. pneumoniae* (223)	57.0 (50.2–63.5)
*S. saprophyticus* (114)	30.7 (22.4–40.0)
*E. coli* (all strains)^a^ (1042)	78.3 (75.7–80.8)

The total number of answers (n) and percentage of correct answers (95% confidence interval) are shown for each bacterial strain. All years are combined for each bacterial strain.

^a^All samples including *E. coli* are combined in *E. coli* (all strains).

**Table 2.** Percentages of correct answers for urine culture.

Urine culture (*n*)	Correct answers (%) (95% CI)
*P. mirabilis* (322)	88.2 (84.2–91.5)
*E. faecalis* (480)	87.7 (84.4–90.5)
*E. cloacae* (148)	78.4 (70.9–84.7)
*K. pneumoniae* (321)	72.9 (67.7–77.7)
*S. saprophyticus* (160)	77.5 (70.2–83.7)
*E. coli* (all strains)^a^ (1412)	87.0 (85.2–88.7)

The total number of answers (n) and percentage of correct answers (95% confidence interval) are shown for each bacterial strain. All years are combined for each bacterial strain.

^a^All samples including *E. coli* are combined in *E. coli* (all strains).

**Table 3.** Percentages of correct answers for susceptibility testing.

Susceptibility testing (*n*)	Correct answers (%) (95% CI)
*P. mirabilis* (res: nitro) (454)	75.8 (71.6–79.6)
*E. faecalis* (res: sulfa + meci) (665)	74.0 (70.5–77.3)
*E. cloacae* (res: ampi + nitro) (219)	67.1 (60.5–73.3)
*K. pneumoniae* (res: ampi + nitro) (441)	69.2 (64.6–73.4)
*S. saprophyticus* (res: meci) (209)	79.9 (73.8–85.1)
*E. coli* (no resistance) (460)	89.8 (86.6–92.4)
*E. coli* (res: cipro) (445)	84.3 (80.5–87.5)
*E. coli* (res: ampi) (238)	79.0 (73.3–84.0)
*E. coli* (res: ampi + meci) (463)	90.7 (87.7–93.2)
*E. coli* (res: ampi + sulfa + trim) (224)	84.8 (79.4–89.3)
*E. coli* (res: sulfa + trim + ampi + cipro) (236)	89.8 (85.2–93.4)

The total number of answers (n) and percentage of correct answers (95% confidence interval) are shown for each bacterial strain. All years are combined for each bacterial strain.

Res: resistant; Nitro: nitrofurantoin; Ampi: ampicillin; Meci: mecilinam; Sulfa: sulfamethizole; Trim: trimethoprim; Cipro: ciprofloxacin.

Table 4 shows the percentages of correct answers for the specific methods used for performing either a urine culture or susceptibility testing. Regarding urine culture, Flexicult had the highest percentage of correct answers (87.6%) followed by chromogenic agar (85.1%) and 2-plate dipslide (85.2%). The lowest percentage of correct answers was found for 3-plate dipslide (72.9%). When looking at susceptibility testing methods, Mueller Hinton agar with tablets (84.6%) had the highest percentage of correct answers followed by Flexicult (77.2%), while Iso-Sensitest with discs had the lowest score (72.9%). Importantly, both Mueller Hinton agar and Iso-Sensitest with tablets resulted in a higher percentage of correct answers than if performed with discs.

**Table 4.** Percentages of correct answers for urine culture and susceptibility testing methods.

Type of urine culture method (*n*)	Correct answers % (95% CI)
Flexicult SSI urinary kit™ (987)	87.6 (85.4–89.6)
Chromogenic agar (1020)	85.1 (82.8–87.2)
2-plate dipslide (427)	85.2 (81.5–88.5)
3-plate dipslide (203)	72.9 (66.2–78.9)
Others (206)	75.2 (68.8–81.0)
**Type of susceptibility testing method (*n*)**	**Correct answers % (95% CI)**
Flexicult SSI urinary kit™ (1109)	77.2 (74.6–79.6)
Mueller Hinton agar with tablets (1918)	84.6 (82.9–86.2)
Mueller Hinton agar with discs (371)	75.5 (70.8–79.8)
Iso-Sensitest with tablets (117)	76.1 (67.3–83.5)
Iso-Sensitest with discs (188)	72.9 (65.9–79.1)
Others (351)	76.6 (71.9–81.0)

The total number of answers (n) and percentage of correct answers (95% confidence interval) are shown for each method. All years and bacterial strains are combined for each method.

The subgroup analysis, exploring the quality of the various methods used for urine culture and susceptibility testing, are presented in Appendixes 1 and 2. It was found that Flexicult had a higher percentage of correct answers for urine culture except for samples including *E. cloacae*, *K. pneumoniae* and *S. saprophyticus*. Mueller Hinton agar with tablets was superior at susceptibility testing for all strains of *E. coli* and *S. saprophyticus* compared to Flexicult. Contrary, Flexicult had a higher percentage of correct answers for *E. cloacae, K. pneumoniae* and *P. mirabilis.*

## Discussion

### Statement of principal findings

In this study, based on 5361 simulated urine samples, microscopy was only performed in about 40% of all examinations. Microscopy has the advantage that the result is ready within a few minutes compared to, for example, a urine culture. However, microscopy requires training, routine and the right equipment. Generally, susceptibility testing was more frequently used than urine culture. The most commonly used methods for urine culture were Flexicult and chromogenic agar. Mueller Hinton agar with tablets – followed by Flexicult – was the most frequently used method for susceptibility testing.

A high quality was found by use of POC tests, when urine samples containing *E. coli* were examined. However, the quality of the microscopic examinations varied a lot, as the general practice staff seemed to have difficulties when evaluating urine samples containing *S. saprophyticus* and *K. pneumoniae*. Concerning *K. pneumoniae*, the low percentage of correct answers did apply for urine culture and susceptibility testing as well. Importantly, higher percentages of correct answers were found when using tablets instead of discs for susceptibility testing.

### Strengths and weaknesses of the study

The strength of this study is a large sample size of simulated urine samples containing the most common uropathogenic bacterial strains observed in Danish general practice. In addition, all participating general practices receiving a fee for performing urinalysis were obligated to attend the quality control programme. Consequently, the risk of selection bias was reduced as not solely practices with a special interest in the management of UTIs participated in the study. However, only data from general practices in the North Denmark Region was included in this study due to availability. So far, no existing literature has described any differences in the quality of urinalysis between general practices in the various Danish regions. However, not all regions in Denmark are supported by the quality control programme MIKAP, and one may assume that the performance quality might be better in regions supported by MIKAP.

Some limitations have to be taken into account when interpreting the results. The simulated single-strain urine samples are not fully representative for urinalysis in general practice, as mixed culture is sometimes seen. Therefore, evaluating the quality of urinalysis based on single-strain cultures might be too optimistic. Furthermore, a standardised amount of bacteria of ≥ 10^5^ per mL does not fully reflect real life urinalysis, as the cut-offs for symptomatic UTI, caused by primary uropathogens (*E. coli, S. saprophyticus)*, are set at ≥ 10^3^ [21]. That is, the performance quality might be overestimated in our study. However, the standardised amount of bacteria and single-strain cultures were an advantage when evaluating and comparing the results and to ensure confluent growth. Importantly, samples without any bacteria were not distributed to participating general practices, and the results of this study cannot be used to evaluate a potential overdiagnosis of UTIs.

Also, urine samples were distributed in containers with boric acid. Previous studies have found that boric acid might interfere with antibiotic sections of Flexicult [16,22]. Furthermore, both elimination and multiplication of especially gram-positive bacteria has been described, and commonly, the boric acid concentration has been found to affect the bacterial strains [15,16,23]. This potential issue was handled by standardised urine and boric acid concentrations. Besides the low percentage of correct answers for microscopy of urine containing *S. saprophyticus,* no consistent findings indicated boric acid to affect gram-positive bacterial strains more than gram-negative bacterial strains.

### Findings in relation to other studies

We found that susceptibility testing was more frequently conducted than urine culture. This finding indicates a problem with diagnosing UTIs in Danish general practice, as only the urine culture contributes with information about the flora composition and bacterial quantification. Importantly, guidelines recommend conducting both urine culture and susceptibility testing for complicated UTI or if the urine dipstick is inconclusive [24]. Consequently, if practices solely conduct susceptibility testing, it might be inadequate information for treatment decision. Additionally, a recent Danish study [25] found that patients with uncomplicated UTI received a more appropriate treatment when only conducting a urine culture compared to performing both a urine culture and susceptibility testing.

We found the results concerning microscopy to be diverging as the percentages of correct answers ranged from 30.7% (*S. saprophyticus*) to 78.3% (*E. coli* (all strains)). This finding is consistent with the findings from a systematic review by Beyer et al. [18] where the clinical validity of microscopy could not be determined. Beyer et al. found a high specificity and a low sensitivity of phase-contrast microscopy, which is the most frequently used type in Denmark [18]. The low sensitivity may be explained by the limitation of phase-contrast microscopy as it is only able to detect quantities of bacteria down to 10^5^ per mL and by < 10^5^ per mL only half of cases will be detected [11,18]. In our study, the difference in percentage of correct answers for microscopy of urine containing *S. saprophyticus* and *E. coli* might be explained by the different appearance of the two bacterial strains. *E. coli* is rod-shaped and motile by which it might be easier to recognise for the practice staff than *S. saprophyticus.*

Furthermore, we found that between 72.9 and 88.2% of all urine culture performed were correct, depending on the type of uropathogen included in the sample. This finding is in line with a study by Holm et al. [15], which showed an agreement of 76% regarding chromogenic agars used for urine culture compared to a reference standard. Importantly, Holm et al. found that 41% of the incorrect results were due to misinterpretation of the culture plate by practice staff [15]. That is, training of general practice staff may raise the accuracy in evaluating urine cultures.

Our study did not provide information whether an incorrect answer for the susceptibility testing was due to wrong interpretation of a sensitive bacterial strain to be resistant or vice versa. However, several studies have found that general practice staff more often misinterprets sensitive bacterial strains as resistant than opposite [26–29]. Consequently, this may lead to overtreatment with second- or third-line antibiotics, even though a first-line antibiotic would have been sufficient [22].

Susceptibility testing with tablets had a higher percentage of correct answers than those performed with discs. Previous studies have presented similar problems with the use of discs and in particular discs containing ampicillin [19,29]. A study by Dornbusch et al. [29] found ampicillin discs to be very sensitive to transportation and emphasised that optimal storage of discs is at –20 °C. Importantly, the discs expire one week after opening of the package which may be exceeded in many practices [30].

### Meaning of the study

We found susceptibility testing to be more frequently used than urine culture, which is inconsistent with the Danish recommendation of urinalysis [10,24]. This finding indicates that education in urinalysis and awareness campaigns for current Danish recommendations could be beneficial in lowering the use of inappropriate antibiotics. Additionally, difficulties in performing urinalysis in general practice should be explored in detail in future qualitative studies taking GPs and general practice staff’s thoughts and considerations into account.

POC tests are already widely used in Danish general practice, but in other European countries, antibiotics are often prescribed empirically based solely on clinical symptoms [22]. For instance, in the UK, only patients who do not respond adequately on first-line antibiotic treatment have urinalysis performed. Considering Denmark having a lower antibiotic consumption compared to other European countries [5], POC tests might be beneficial to reduce inappropriate antibiotic prescription.

## Geolocation information

The North Denmark Region, Denmark.

## Appendix 1.

### Percentages of correct answers for urine culture subdivided in methods

**Table:**

Type of urine culture method (n)	Correct answers (%)
*P. mirabilis*	
Flexicult SSI urinary kit™ (113)	95.6
Chromogenic agar (114)	86.8
2-plate dipslide (50)	88.0
3-plate dipslide (23)	82.6
Others (22)	54.5
*E. faecalis*	
Flexicult SSI urinary kit™ (168)	95.8
Chromogenic agar (173)	91.3
2-plate dipslide (68)	76.5
3-plate dipslide (38)	63.2
Others (33)	72.7
*E. cloacae*	
Flexicult SSI urinary kit™ (49)	79.6
Chromogenic agar (52)	69.2
2-plate dipslide (22)	95.5
3-plate dipslide (9)	88.9
Others (16)	75.0
*K. pneumoniae*	
Flexicult SSI urinary kit™ (119)	67.2
Chromogenic agar (116)	76.2
2-plate dipslide (43)	83.7
3-plate dipslide (19)	63.2
Others (24)	58.3
*S. saprophyticus*	
Flexicult SSI urinary kit™ (64)	70.3
Chromogenic agar (54)	77.8
2-plate dipslide (22)	90.9
3-plate dipslide (9)	88.9
Others (11)	72.7
*E. coli* (all strains)^a^	
Flexicult SSI urinary kit™ (474)	91.6
Chromogenic agar (511)	85.9
2-plate dipslide (222)	84.6
3-plate dipslide (105)	73.4
Others (100)	80.9

The total number of answers (n) and percentage of correct answers are shown for each method. All years are combined for each method.

^a^All samples including *E. coli* are combined in *E. coli* (all strains).

## Appendix 2.

### Percentages of correct answers for susceptibility testing subdivided in methods

**Table:**

Type of susceptibility testing method (n)	Correct answers (%)
*P. mirabilis* (res: nitro)	
Flexicult SSI urinary kit™ (122)	91.0
Mueller Hinton agar with tablets (224)	78.1
Mueller Hinton agar with discs (44)	59.1
Iso-Sensitest with tablets (13)	53.8
Iso-Sensitest with discs (27)	33.3
Others (24)	66.7
*E. faecalis* (res: sulfa + meci)	
Flexicult SSI urinary kit™ (197)	69.0
Mueller Hinton agar with tablets (299)	76.3
Mueller Hinton agar with discs (63)	81.0
Iso-Sensitest with tablets (16)	81.3
Iso-Sensitest with discs (34)	70.6
Others (56)	73.2
*E. cloacae* (res: ampi + nitro)	
Flexicult SSI urinary kit™ (62)	82.3
Mueller Hinton agar with tablets (96)	66.7
Mueller Hinton agar with discs (20)	65.0
Iso-Sensitest with tablets (8)	37.5
Iso-Sensitest with discs (8)	25.0
Others (25)	56.0
*K. pneumoniae* (res: ampi + nitro)	
Flexicult SSI urinary kit™ (122)	73.8
Mueller Hinton agar with tablets (218)	69.7
Mueller Hinton agar with discs (37)	64.9
Iso-Sensitest with tablets (15)	46.7
Iso-Sensitest with discs (8)	50.0
Others (41)	68.3
*S. saprophyticus* (res: meci)	
Flexicult SSI urinary kit™ (62)	74.2
Mueller Hinton agar with tablets (103)	87.4
Mueller Hinton agar with discs (21)	61.9
Iso-Sensitest with tablets (2)	100.0
Iso-Sensitest with discs (6)	66.7
Others (15)	80.0
*E. coli* (no resistance)	
Flexicult SSI urinary kit™ (123)	87.0
Mueller Hinton agar with tablets (209)	92.8
Mueller Hinton agar with discs (45)	86.7
Iso-Sensitest with tablets (13)	92.3
Iso-Sensitest with discs (31)	93.5
Others (39)	79.5
*E. coli* (res: cipro)	
Flexicult SSI urinary kit™ (117)	70.1
Mueller Hinton agar with tablets (217)	93.1
Mueller Hinton agar with discs (40)	80.0
Iso-Sensitest with tablets (10)	90.0
Iso-Sensitest with discs (15)	80.0
Others (46)	82.6
*E. coli* (res: ampi)	
Flexicult SSI urinary kit™ (62)	56.5
Mueller Hinton agar with tablets (111)	93.7
Mueller Hinton agar with discs (19)	68.4
Iso-Sensitest with tablets (9)	88.9
Iso-Sensitest with discs (15)	73.3
Others (23)	73.9
*E. coli* (res: ampi + meci)	
Flexicult SSI urinary kit™ (126)	81.7
Mueller Hinton agar with tablets (225)	96.0
Mueller Hinton agar with discs (41)	87.8
Iso-Sensitest with tablets (16)	87.5
Iso-Sensitest with discs (23)	100.0
Others (31)	90.3
*E. coli* (res: ampi + sulfa + trim)	
Flexicult SSI urinary kit™ (55)	81.8
Mueller Hinton agar with tablets (106)	89.6
Mueller Hinton agar with discs (22)	68.2
Iso-Sensitest with tablets (6)	100.0
Iso-Sensitest with discs (7)	71.4
Others (28)	85.7
*E. coli* (res: sulfa + trim + ampi + cipro)	
Flexicult SSI urinary kit™ (61)	82.0
Mueller Hinton agar with tablets (110)	92.7
Mueller Hinton agar with discs (19)	94.7
Iso-Sensitest with tablets (9)	88.9
Iso-Sensitest with discs (14)	100.0
Others (23)	87.0

The total number of answers (n) and percentage of correct answers are shown for each method. All years are combined for each method.

Res: resistant; Nitro: nitrofurantoin; Ampi: ampicillin; Meci: mecilinam; Sulfa: sulfamethizole; Trim: trimethoprim; Cipro: ciprofloxacin.
